# Enhancing Photocurrent of Radially Polarized Ferroelectric BaTiO_3_ Materials by Ferro-Pyro-Phototronic Effect

**DOI:** 10.1016/j.isci.2018.04.016

**Published:** 2018-04-25

**Authors:** Kun Zhao, Bangsen Ouyang, Ya Yang

**Affiliations:** 1 CAS Center for Excellence in Nanoscience, Beijing Institute of Nanoenergy and Nanosystems, Chinese Academy of Sciences, Beijing 100083, P. R. China; 2 School of Nanoscience and Technology, University of Chinese Academy of Sciences, Beijing 100049, P. R. China

**Keywords:** Electrical Materials, Energy Materials, Nanotechnology Fabrication

## Abstract

The pyro-phototronic effect has been utilized to modulate photoexcited carriers, to enhance the photocurrent in semiconducting nanomaterials. However, most of these materials have low pyroelectric performances. Using radially polarized ferroelectric BaTiO_3_ materials with a pyroelectric coefficient of about 16 nC/cm^2^K, we report a dramatic photocurrent enhancement due to ferro-pyro-phototronic effect. The fabricated device enables a fast sensing of 365-nm light illumination with a response time of 0.5 s at the rising edge, where the output current-time curve displays a sharp peak followed by a stable plateau. By applying a heating temperature variation, the output current peak can be enhanced by more than 30 times under a light intensity of 0.6 mW/cm^2^. Moreover, the stable current plateau can be enhanced by 23% after utilizing a cooling temperature variation, which can be well explained by ferro-pyro-phototronic-effect-induced energy band bending.

## Highlights

• We present a new method to realize photocurrent enhancement in BaTiO_3_ materials
• The fabricated device enables fast sensing of 365-nm light illumination
• The principle relies on the ferro-pyro-phototronic effect of BaTiO_3_

## Graphical Abstract

**Figure:**
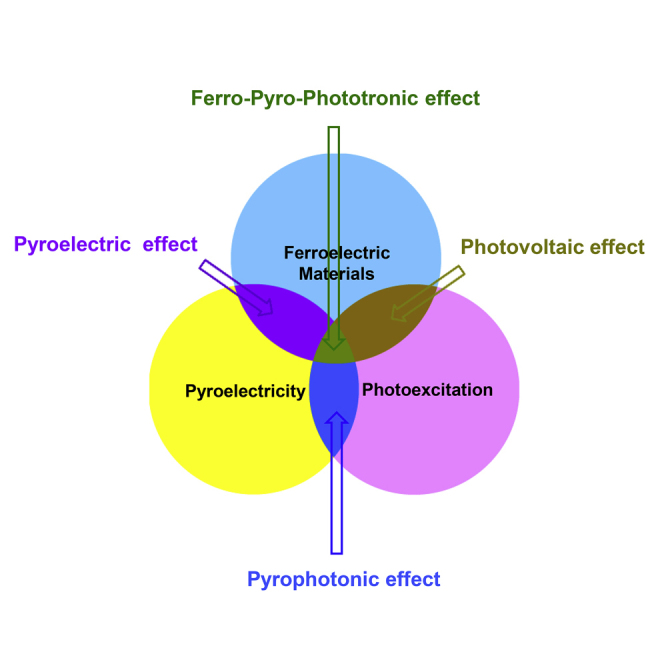

## Introduction

**Figure 1:**
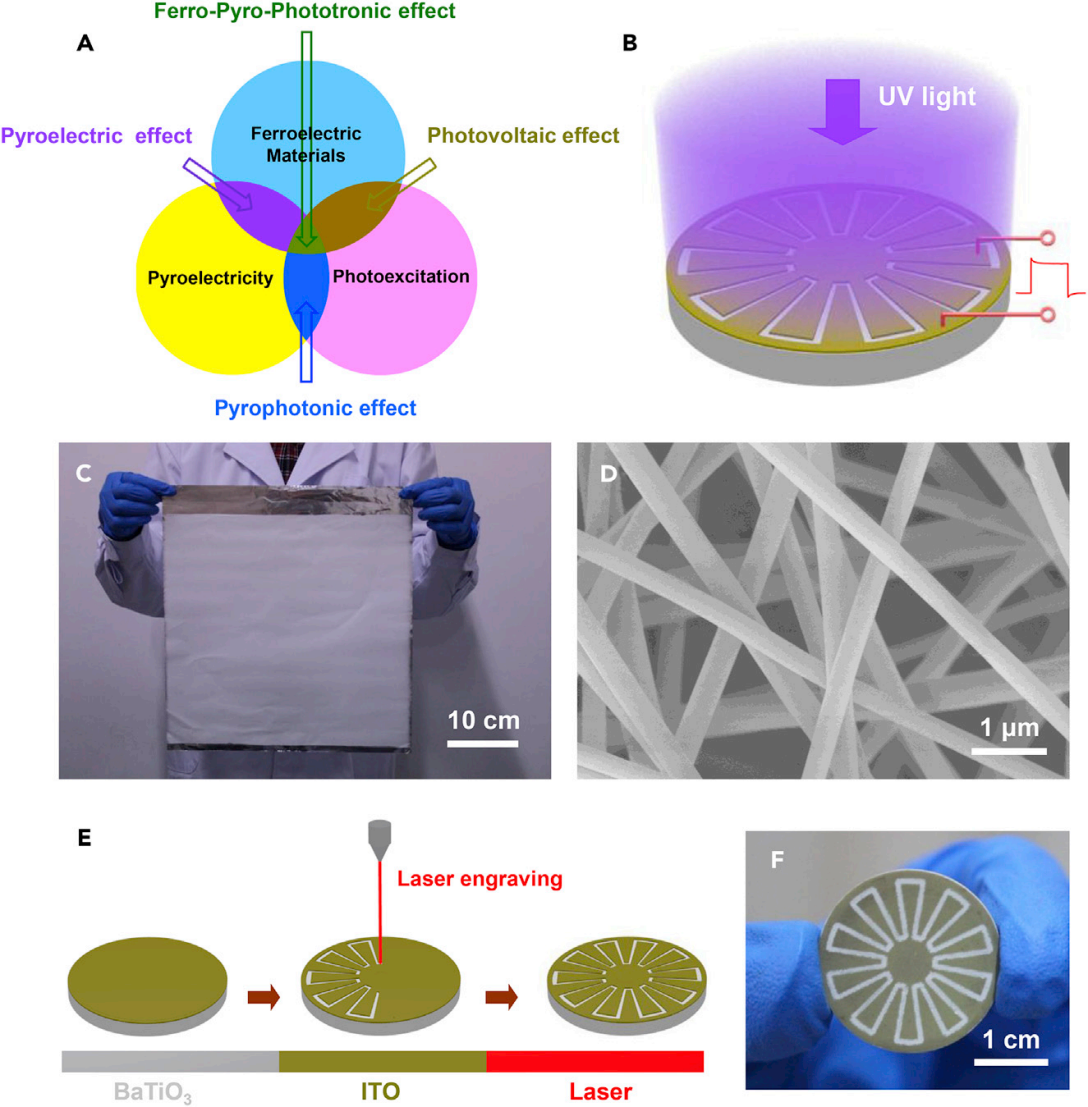
Fabrication of Ferroelectric BaTiO_3_ Device (A) Schematic diagram of ferro-pyro-phototronic effect showing the three-way coupling among pyroelectricity, photoexcitation, and ferroelectric materials. (B) Schematic diagram of the fabricated device. (C) Photograph of BaTiO_3_ precursor nanowires with a large area. (D) Scanning electron microscopic image of the BaTiO_3_ precursor nanowires. (E) Schematic diagram of the laser engraving process on the surface of device. (F) Photograph of the fabricated device. See also Figure S1.

Over the past decade, investigations of photocurrent in ferroelectric materials have attracted particular attention related to the use of polarization-induced inner electric field to achieve the effective separation of photo-generated charges (Grinberg et al., 2013, Choi et al., 2009, Cao et al., 2012). Recent advances about enhancement of photocurrent in ferroelectric materials include large stain gradients (Chu et al., 2015), nanostructures on the ferroelectric material surface (Spanier et al., 2016), and electric field control over domain structures (Yang et al., 2010). There has been no report about temperature variation dependence of photocurrent in ferroelectric materials, despite its importance for developing light-heating coupled device applications. Pyro-phototronic effect is based on the related three-way coupling among semiconductors, pyroelectricity, and photoexitation, which has been utilized to enhance interfacial charge transfer for improving the photovoltaic performances of ZnO nanowire-based solar cells and photodetectors (Zhang et al., 2016, Wang et al., 2017). However, it has remained a challenge to achieve larger modulation of photocurrent in the reported semiconducting ZnO nanowire since it has a low pyroelectric performance with a corresponding pyroelectric coefficient of about 1.5 nC/cm^2^K (Yang et al., 2012). As a result, it is necessary to exploit the other pyroelectric materials for enabling the next generation of high-performance pyroelectric-photovoltaic devices. Some ferroelectric materials can have much better pyroelectric performance than ZnO nanowires, such as Pb(Zr,Ti)O_3_ (Vats et al., 2016, Guo et al., 2007, Ko et al., 2016, Zhang et al., 2017), BaTiO_3_ (Ban and Alpay, 2003), and LiNbO_3_ (Savage, 1966, Weis and Gaylord, 1985), where BaTiO_3_ is a prototypical lead-free ferroelectric material with a moderate Curie temperature (T_c_ = 120°C) (Dubourdieu et al., 2013). Moreover, these ferroelectric materials also have dramatic photovoltaic performances under short-wavelength light illuminations (Ichiki et al., 2004, Paillard et al., 2016). Instead of using semiconductor materials, a pyroelectric effect-induced inner electric field in ferroelectric materials can be also utilized to enhance the separation of electrons and holes induced by light illumination. Herein, the related three-way coupling among ferroelectric materials, pyroelectricity, and photoexcitation can be termed as the ferro-pyro-phototronic effect, as illustrated in Figure 1A. The fundamental issues of the pyroelectricity dependence of photocurrent in ferroelectric materials, and of the possible enhancement of photocurrent by heating or cooling temperature variations, become crucial.

In this work, we utilized the temperature-variation-induced ferro-pyro-phototronic effect in radially polarized BaTiO_3_ film with a pyroelectric coefficient of about 16 nC/cm^2^K to enhance the photocurrent of the device under 365-nm ultraviolet (UV) light illumination. The photocurrent of polarized BaTiO_3_ film exhibits a sharp current peak followed by a stable current plateau as well. Under a light intensity of 0.6 mW/cm^2^, the output current peak can be significantly enhanced by more than 30 times by applying an external heating temperature variation. Moreover, the stable photocurrent plateau can be increased by 23% after using an external cooling temperature variation, which can be well explained by ferro-pyro-phototronic-effect-induced energy band bending at the interfaces between BaTiO_3_ and indium tin oxide (ITO) electrodes. These results present a major step toward the exploitation of pyroelectric-photovoltaic devices for energy scavenging and self-powered sensing applications.

## Results

### Fabrication of Radially Polarized BaTiO_3_ Device

Figure 1B is a schematic diagram of a radially polarized BaTiO_3_ device under UV light illumination, wherein the transparent ITO on the surface of a BaTiO_3_ film was utilized as electrodes. The BaTiO_3_ precursor nanowires were synthesized by an electro-spinning method with a large area of 32 cm × 32 cm, as displayed in Figure 1C. The obtained BaTiO_3_ precursor nanowires have diameters less than 500 nm (Figures 1D and S1A). To remove the organic materials on the surfaces of BaTiO_3_ nanowires, the fabricated samples were sintered at 1,200°C in 6 hr. The corresponding X-ray diffraction patterns (Figure S1B) indicate that the BaTiO_3_ nanomaterials after being sintered at 1,200°C have an orthorhombic phase. The BaTiO_3_ precursor nanowires after being sintered are changed to BaTiO_3_ nanoparticles with diameters ranging from 300 nm to 1.5 μm (Figures S1C and S1D). Good dispersion of the BaTiO_3_ nanoparticles can be found after an effective mechanical lapping (Figures S1E and S1F). The BaTiO_3_ film can be obtained by sintering the extruded nanowires-polyvinyl alcohol (PVA) mixtures under a pressure of 9 MPa; the corresponding SEM image of the BaTiO_3_ film exhibits a good connection between the adjacent BaTiO_3_ particles. A laser engraving method was utilized to fabricate planar ITO electrodes on the surface of BaTiO_3_ film (Figure 1E). A high voltage was applied between the two planar ITO electrodes to realize the radial polarization of the BaTiO_3_ film. Figure 1F is a photograph of the fabricated device with 10 pairs of engraved ITO electrodes, the device having a diameter of about 25 mm and a thickness of about 1 mm.

### Photovoltaic and Pyroelectric Performances of Radially Polarized BaTiO_3_ Device

**Figure 2:**
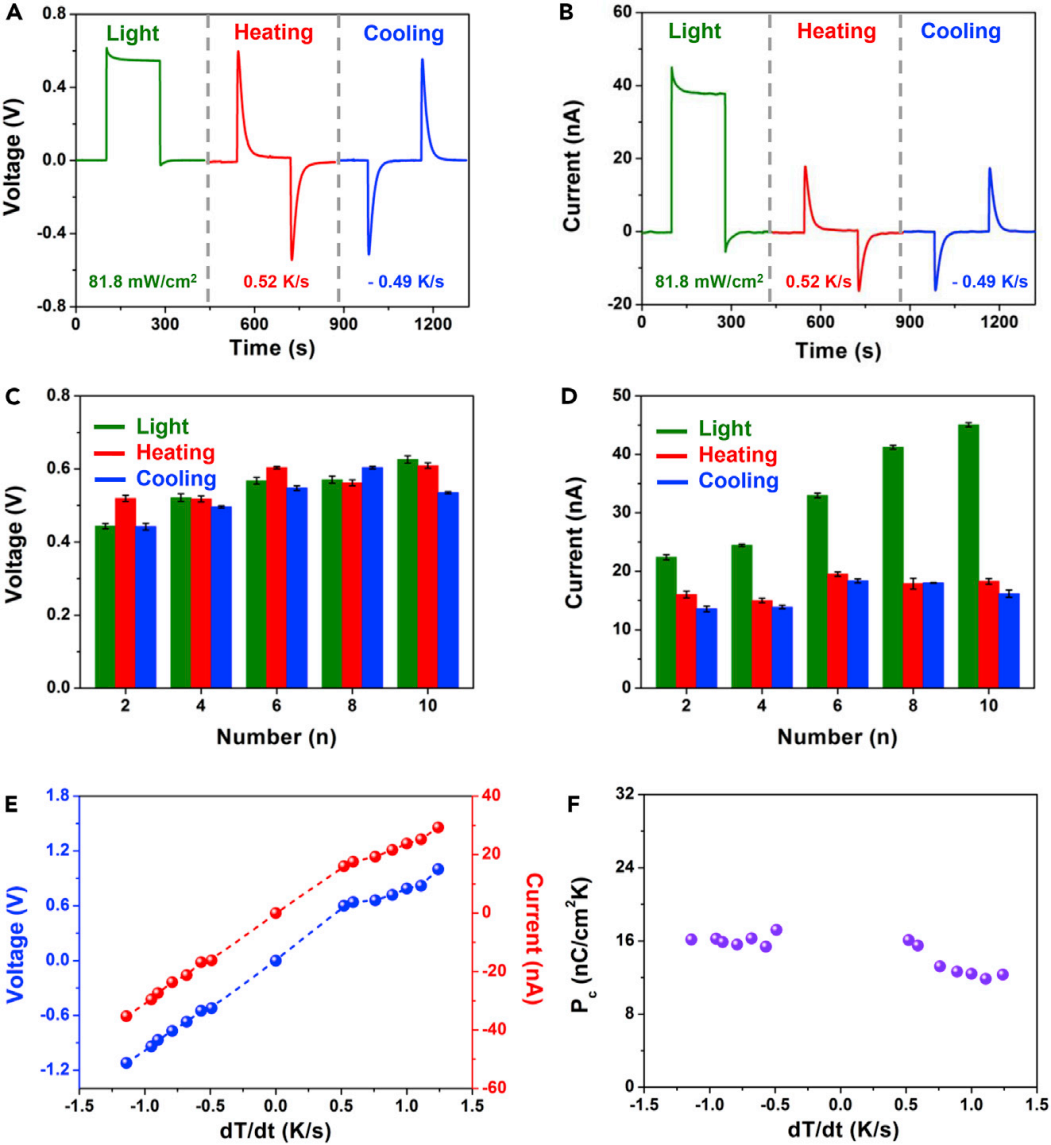
Photovoltaic and Pyroelectric Performances of Radially Polarized BaTiO_3_ Device (A and B) Measured output voltage (A) and current (B) signals of the device (N = 10) under individual light illumination and heating and cooling conditions. (C and D) Comparison of the measured output voltage (C) and current peak (D) signals of the different devices with different ITO electrode pair numbers. (E) Measured output voltage and current peak signals of the device (N = 10) under different temperature variation rates. (F) Calculated pyroelectric current coefficients of the device (N = 10) under different temperature variation rates. See also Figures S2–S11.

Figures 2A and 2B illustrate the measured output voltage and current signals of the fabricated device (Figure 1F) under UV light illumination, heating, and cooling conditions, respectively. Under a UV light illumination intensity of 81.8 mW/cm^2^, the device delivers a DC output voltage peak of about 0.62 V and then a voltage plateau of about 0.55 V. The corresponding output current peak and plateau are about 45 nA and 38 nA, respectively. Owing to the pyroelectric effect of BaTiO_3_, the device can produce AC output voltage/current peaks under heating or cooling conditions. Under a heating temperature variation rate of 0.52 K/s, the output voltage and current peaks are about 0.6 V and 18.3 nA, respectively. Reversed output voltage and current signals can be observed when a cooling temperature variation was applied. The effect of ITO electrode pair number on the performance of device has been also investigated by designing different electrode pair numbers ranging from 2 to 10 (Figure S2). The comparisons of the output voltage and current peaks of these devices are displayed in Figures 2C and 2D by the analysis of measured voltage/current-time data (Figures S3–S6). A slight increase of output voltage under light illumination or heating/cooling conditions can be observed (Figure 2C). Moreover, the output photovoltaic current exhibits a dramatic increase from 18.3 nA to 38.1 nA on increasing the ITO electrode pair number, although a small variation can be seen for pyroelectric current.

The pyroelectric performance of radially polarized BaTiO_3_ device with 10 ITO electrode pairs was systematically investigated by measuring the output current and voltage signals under different temperature variation rates. The corresponding temperature variation distribution and temperature-time curves can be recorded by using an infrared thermometer (Figures S7 and S8). The output voltage and current signals of the device were measured under different temperature variation rates (Figures S9 and S10). Under a cooling temperature variation rate of −1.14 K/s, the output current is about −35.27 nA, whereas the output current is about 29.26 nA under a heating temperature variation rate of 1.25 K/s. By analysis of these data, it can be found that both the output voltage and current signals scale linearly with the temperature variation rate, as illustrated in Figure 2E. The pyroelectric current coefficient can be described as $P_{C}=I/\left(A\cdot dT/dt\right)$, where *I* is the pyroelectric current, *A* is the electrode area, and *dT/dt* is the temperature variation rate (Lang, 2005). Under the cooling temperature variation conditions, the corresponding pyroelectric current coefficient can be calculated to be about 16 nC/cm^2^K (Figure 2F), which is 10 times larger than that of the reported ZnO nanowires (1.5 nC/cm^2^K). Moreover, it is 7.6 times larger than that of the reported Ag/BTO/Ag device with a vertical structure (2.1 nC/cm^2^K) (Ma and Yang, 2017). The pyroelectric current coefficient decreases with increasing temperature, which is associated with a lower polarization intensity under a higher temperature. Moreover, the BaTiO_3_ has a Curie temperature of 120°C (Dubourdieu et al., 2013), and there is no spontaneous polarization if the temperature is larger than 120°C. Thus the pyroelectric current coefficients exhibit a slight decrease under the heating temperature variation conditions, as displayed in Figure 2F. Meanwhile, we calculated the pyroelectric current coefficient of the device with different numbers of ITO electrodes, showing that the electrode effective area of the device decreases with an increase in the number of electrodes. The pyroelectric current coefficient exhibits a slight increase with an increase in the number of ITO electrodes under the same temperature variations (Figure S11).

### Photocurrent Enhancement of Radially Polarized BaTiO_3_ Device

**Figure 3:**
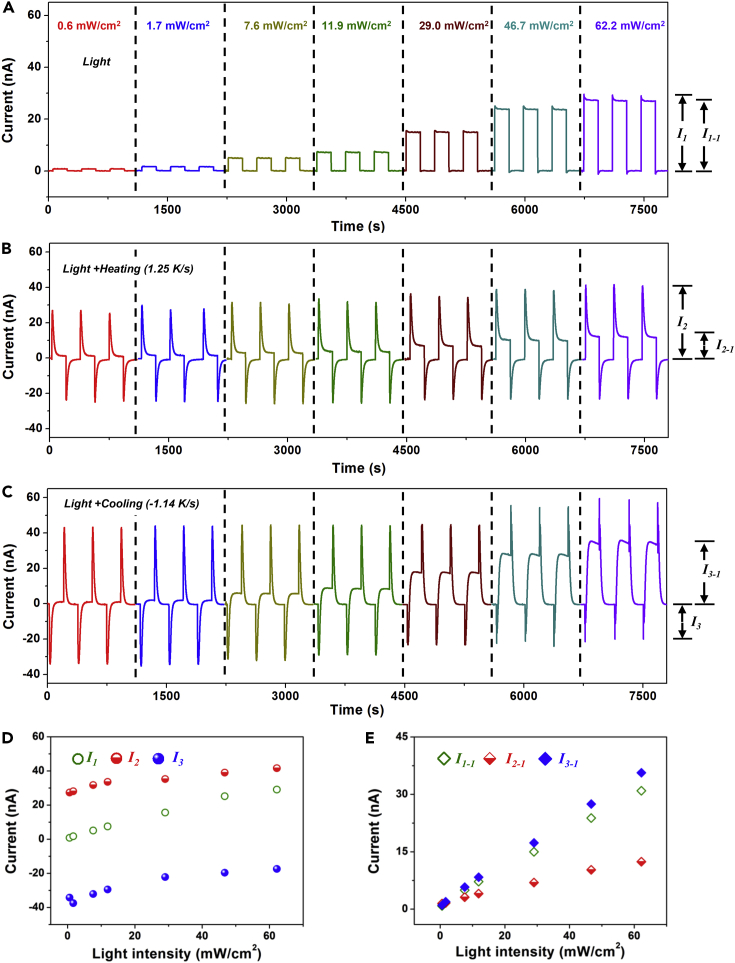
Photocurrent Enhancement of Radially Polarized BaTiO_3_ Device (A) Measured output current signals of the device under 365-nm UV illumination with different light intensities, where *I*_*1*_ is the current peak, *I*_*1-1*_ is the current plateau. (B and C) Measured output current signals of the device under simultaneous light illumination with different intensities and temperature variations with a heating temperature change rate of 1.25 K/s (B) and a cooling temperature change rate of −1.08 K/s (C), where *I*_*2*_ and *I*_*3*_ are the current peaks and *I*_*2-1*_ and *I*_*3-1*_ are the current plateaus. (D and E) Comparison of the measured output current peak (D) and plateau (E) signals of the devices under the different light intensities. See also Figures S12–S15.

The heating-induced pyroelectric effect has been utilized to enhance the UV light response. Since the silver electrode is opaque to UV light, the fabricated Ag/BTO/Ag device exhibited only pyroelectric effect and did not show photovoltaic effect (Ma and Yang, 2017). Ferro-pyro-phototronic effect utilizes a temperature variation-induced pyroelectric electric field to modulate photoexcited carriers for enhancing photocurrent in ferroelectric materials owing to the coupling among ferroelectric material, pyroelectricity, and phtoexcitation. BaTiO_3_ is a ceramic material with a low carrier concentration and a high pyroelectric coefficient. There is no obvious screening effect of photo-excited carriers on the pyroelectric polarization of BaTiO_3_. As a comparison, ZnO nanowire is a semiconductor with relatively higher carrier concentration and lower pyroelectric coefficient (Yang et al., 2012), which may result in a significant screening effect. Figure 3A depicts the output current signals of the device under different UV light illuminations, where the output current increases with increasing intensity of UV light. As presented in Figure 3B, a sharp current peak followed by a stable plateau can be seen under simultaneous light illumination and heating temperature variation. The current peak increases with increasing light intensity, whereas the stable current plateau is smaller than that of individual UV light illumination. Figure 3C illustrates the output current data under simultaneous light illumination and cooling temperature variation, indicating that the current plateau can be effectively enhanced by the cooling temperature variation. The corresponding output voltage signals of the devices under individual UV light illumination and simultaneous light-temperature variation also exhibit similar changing tendency (Figure S12). To compare the change of photocurrent under different conditions, the output current peak signals of the device in Figures 3A–3C are displayed in Figure 3D, indicating that a heating temperature variation can be utilized to enhance the output current peak signals of the device. Under a light intensity of about 0.6 mW/cm^2^, the photocurrent of the device under simultaneous UV light illumination and heating temperature variation can be enhanced by more than 30 times when compared with the photocurrent of the device under individual light illumination (Figure S13). The enhancement ratio of photocurrent decreases with increasing light intensity. In Figure 3D, the current value of I_3_-I_1_ (light + cooling) is larger than that of I_2_-I_1_ (light + heating), which is associated with the larger pyroelectric coefficients under cooling conditions (Figure 2F). The output photocurrent plateau signals of the devices under the different conditions are displayed in Figure 3E, indicating that a cooling temperature variation can be used to enhance the photocurrent plateau. The enhancement ratio increases with decreasing light illumination intensity (Figure S13B), whereas the corresponding photocurrent can be enhanced by about 23% under a light intensity of 0.6 mW/cm^2^. Because both peak current and plateau current signals generated by the light illumination are small at the low light intensity (0%–15%), the current peak and plateau enhancement ratios are large. With the increase of the light intensity, both peak current and plateau current signals generated by the light illumination are slowly increased, so the enhancement rate tends to be saturated.

To evaluate the UV light detection sensitivity of the device, both the responsivity *R* = *I*_*ph*_*/(PS)* and specific detectivity *D*^∗^ = *R*/(2e·*I*_dark_/*S*)^0.5^ were calculated by using the experiential data in Figure 3D, where *I*_*ph*_ is the photocurrent, *P* is the incident light intensity, *S* is the effective irradiated area, and *e* is the electronic charge (Zheng et al., 2016, Dhar et al., 2016). Under a light intensity of 0.6 mW/cm^2^, the calculated responsivity values of the device under individual light illumination and simultaneous light-heating conditions are 2.3×10^−7^ A/W and 71.7×10^−7^ A/W, respectively (Figure S13C). They are 2.8 and 117.5 times larger than those of the reported Ag/BTO/Ag device in the coupled lighting and heating conditions (6.1 ×10^−8^ A/W) (Ma and Yang, 2017). The corresponding specific detectivity values of the device under individual light illumination and simultaneous light-heating conditions are 11.9 Jones and 378.8 Jones, respectively. When compared with individual light illumination, the heating-induced ferro-pyro-phototronic effect leads to a huge enhancement of both the responsivity and the specific detectivity by more than 30 times under a light intensity of 0.6 mW/cm^2^, although the enhancement ratio can be decreased with increasing light intensity (Figure S13D). We also investigated the response time of the device to UV light illumination (Figure S14), clearly showing that the fabricated device exhibits a good photoresponse component with a rise time of 0.5 s and a fall time of 0.2 s, where the time was calculated between 10% and 90% of the photocurrent signal when the UV light was turned on and off, respectively. We also measured the stability of device under more than 240 cycles in 24 hr (Figure S15), where a constant output voltage peak of about 0.62 V in all cycles indicates an excellent stability for the fabricated device.

### The Mechanism for the Enhancement of Photocurrent Plateau

**Figure 4:**
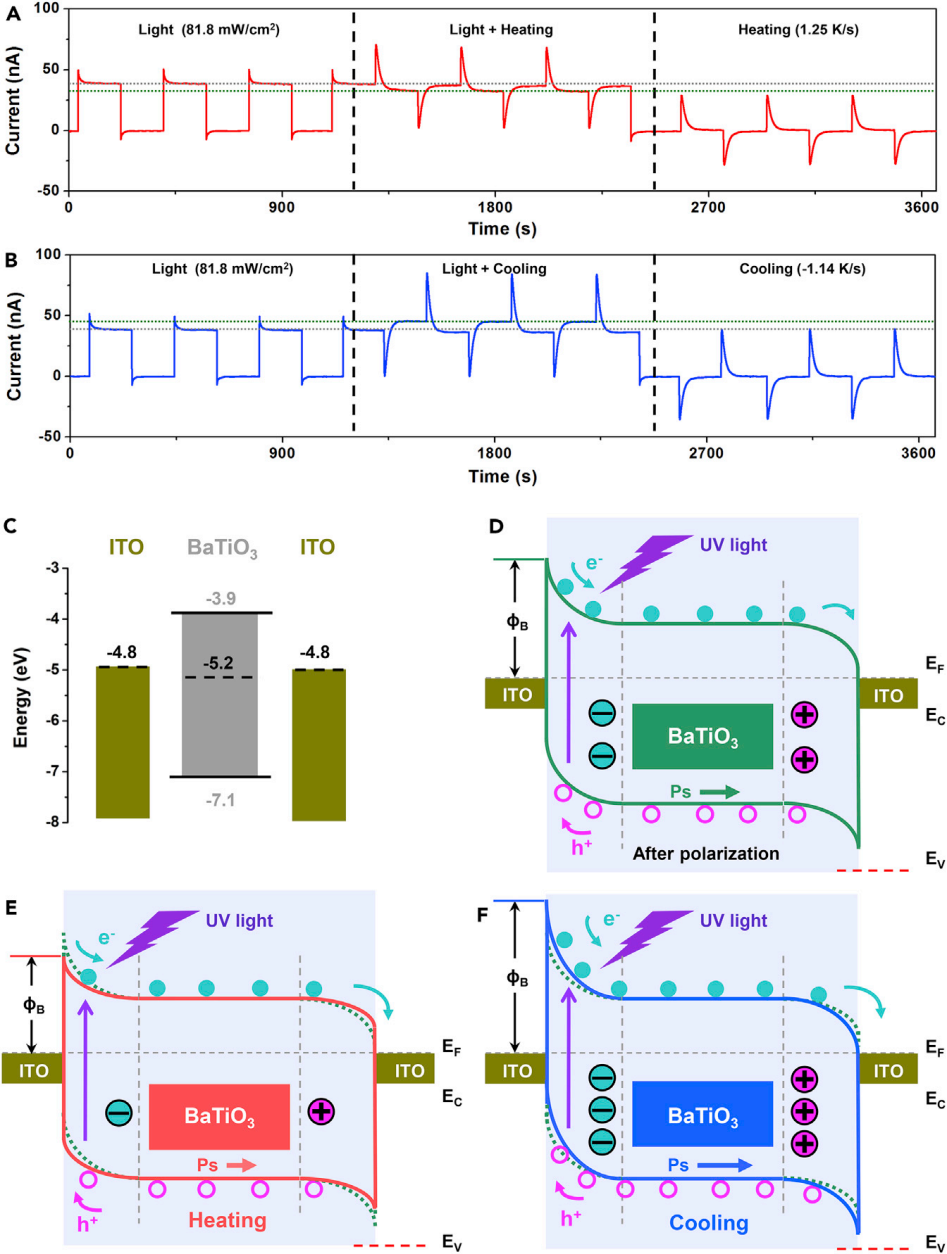
The Mechanism for the Enhancement of Photocurrent Plateau (A and B) Measured output current signals of the device under the individual light illumination, the individual heating (A) or cooling (B) temperature variations, and the simultaneous light illumination and temperature variations. (C) Energy-level diagram showing the valence and conduction energies of each component material in the BaTiO_3_ device. (D–F) Schematic illustrations of the energy band structures of the device (D) under 365-nm UV illumination after polarization of the device, (E) under simultaneous 365-nm UV illumination and heating conditions after polarization of the device, and (F) under simultaneous 365-nm illumination and cooling conditions after polarization. See also Figure S16.

To identify the effect of ferro-pyro-phototronic effect on the photocurrent plateau further, we measured photocurrent signals of the device under individual light illumination (81.8 mW/cm^2^), heating (1.25 K/s), and simultaneous light-heating conditions, as displayed in Figure 4A. It can be seen that the photocurrent under simultaneous light-heating condition is not simple superposition of the photocurrent signals under individual light and heating conditions. When compared with individual light illumination, the photocurrent plateau can be decreased due to the coupling of light and heating. When compared with individual light and cooling conditions, the coupling enhancement (“1 + 1 > 2”) of photocurrent plateau can be clearly observed when simultaneous light and cooling were applied on the device, as illustrated in Figure 4B. The output voltage signals of the device exhibit a similar change (Figure S16). However, according to Figures 4A and 4B, the illumination peak current is 50 nA and the pyroelectric peak currents are 29.3 nA and −36 nA under the heating and cooling conditions, respectively. The measured total peak current values are 71 nA (<50 nA + 29.3 nA) and 0.2 nA (<50 nA − 36 nA) under light + heating and light + cooling conditions, respectively. Therefore the relationship between the total peak current and the individual current is not a simple additive one.

This behavior for the enhancement of photocurrent plateau in Figure 4B can be well understood due to the effect of ferro-pyro-phototronic effect on the energy band structures of the device, as depicted in Figures 4C–4F. The energy-level diagram showing the valence and conduction energies of each component material in the BaTiO_3_ device is depicted in Figure 4C. The device has a structure of ITO/BaTiO_3_/ITO, which can be regarded as a BaTiO_3_ layer sandwiched between two back-to-back Schottky barriers at the interfaces. After the BaTiO_3_ layer was polarized, the negative and positive polarized charges can increase and decrease the Schottky barrier height at the two BaTiO_3_/ITO interfaces, respectively. As displayed in Figure 4D, the UV light illumination-induced electron and hole pairs can be effectively separated by the left Schottky barrier to produce the observed photocurrent signals. When the device is heating, the polarized charges of the BaTiO_3_ layer can be decreased, resulting in the reduction of the left Schottky barrier height. Moreover, the right Schottky barrier height can be increased due to the decrease of positive polarized charges in BaTiO_3_, as depicted in Figure 4E. The reduction of left Schottky barrier height will weaken the effective separation of light-induced electron-hole pairs, and the increased Schottky barrier height at the right interface is detrimental to the flow of electrons from BaTiO_3_ to the right ITO electrode, resulting in the decrease of photocurrent plateau in Figure 4A. However, when the device is cooling, the polarized charges of BaTiO_3_ layer can be increased, resulting in the increase of the left Schottky barrier height, which is more effective to separate the light-induced electron-hole pairs, as illustrated in Figure 4F. The decrease of right Schottky barrier height will further promote the flow of electrons from BaTiO_3_ to the right ITO electrode. As a result, the photocurrent plateau can be effectively enhanced by the coupling of light and cooling in the device.

## Discussion

In summary, we have demonstrated how to utilize ferro-pyro-phototronic effect to realize a dramatic photocurrent enhancement in radially polarized ferroelectric BaTiO_3_ materials with a pyroelectric coefficient of about 16 nC/cm^2^K by coupling light illumination and cooling temperature variation. Under 365-nm light illumination, the fabricated BaTiO_3_ device delivers a sharp current peak followed by a stable current plateau as well. When compared with individual light illumination, the output current peak signals of the device can be enhanced by more than 30 times by using the coupling of light and heating under a light intensity of 0.6 mW/cm^2^. Moreover, the stable output current plateau can be enhanced by 23% due to the coupling of light and cooling, which can be well explained by ferro-pyro-phototronic-effect-induced band bending at the interfaces of BaTiO_3_ and ITO electrodes. Our results reveal the role of ferro-pyro-phototronic effect in ferroelectric BaTiO_3_ photodetectors, which has potential applications in improving the output performances of optoelectronic devices based on ferroelectric materials such as solar cells and photodetectors.

## Methods

All methods can be found in the accompanying Transparent Methods supplemental file.

## Supplemental Information

Document S1. Transparent Methods and Figures S1–S16
