# Supplementary material for: Enhancing Photocurrent of Radially Polarized Ferroelectric BaTiO3 Materials by Ferro-Pyro-Phototronic Effect
Source: iScience. 2018 Apr 25;3:208–16. doi: 10.1016/j.isci.2018.04.016 (PMC6137385; doi:10.1016/j.isci.2018.04.016)
Supplement: Document S1. Transparent Methods and Figures S1–S16 [file mmc1.pdf]

ISCI, Volume *3*

Supplemental Information

Enhancing Photocurrent of Radially Polarized Ferroelectric $BaTiO_3$ Materials by Ferro-Pyro-Phototronic Effect

Kun Zhao, Bangsen Ouyang, and Ya Yang

SUPPLEMENTAL FIGURES

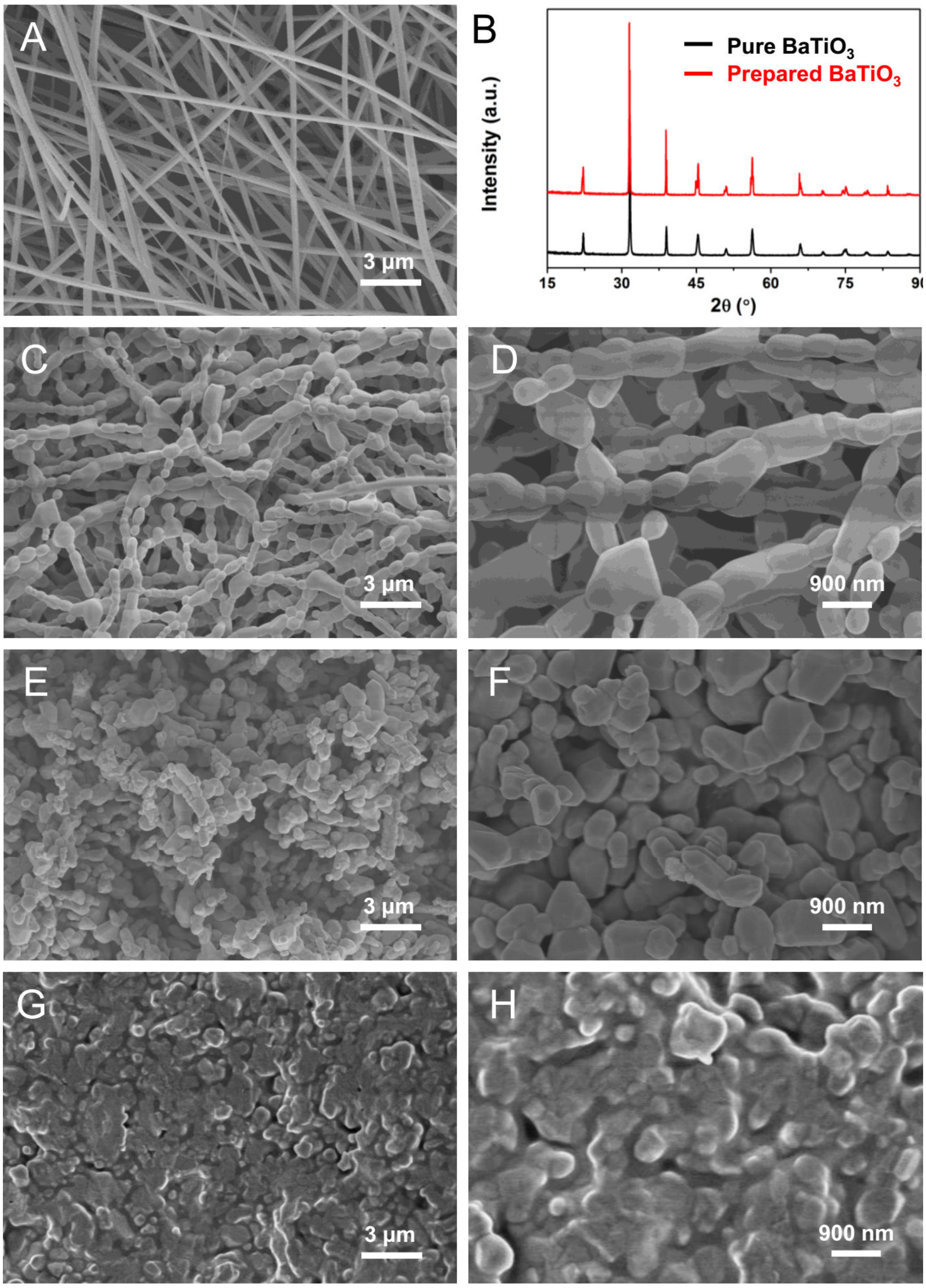

Figure S1: Characterization of $BaTiO_3$ precursor nanowires, nano-$BaTiO_3$ and $BaTiO_3$ films, related to Figure 1.

(A) SEM image of the $BaTiO_3$ precursor nanowires with the low magnification.

(B) XRD patterns of the prepared and pure $BaTiO_3$ nanoparticles.

(C and D) SEM images of the obtained $BaTiO_3$ nanowires with the low (C) and high (D) magnifications.

(E and F) SEM images of the obtained $BaTiO_3$ nanoparticles with the low (E) and high (F) magnifications.

(G and H) SEM images of the $BaTiO_3$ films sintered at 1200 ºC with the low (G) and high (H) magnifications.

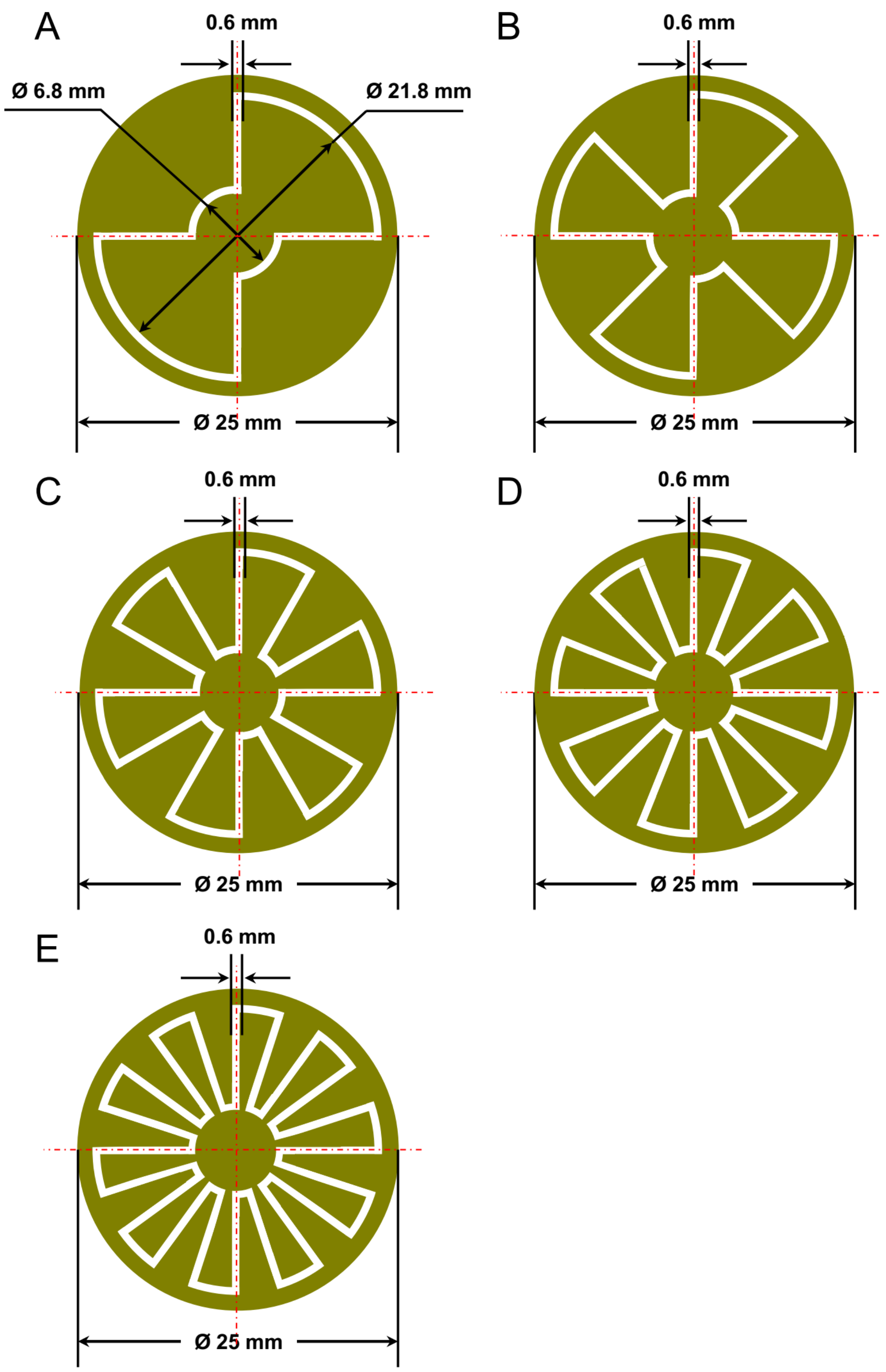

Figure S2: Schematic illustrations of the device's size, related to Figure 2.
(A-E) The device's size with the engraved ITO electrode number of 2 (A), 4 (B), 6 (C), 8 (D) and 10 (E).

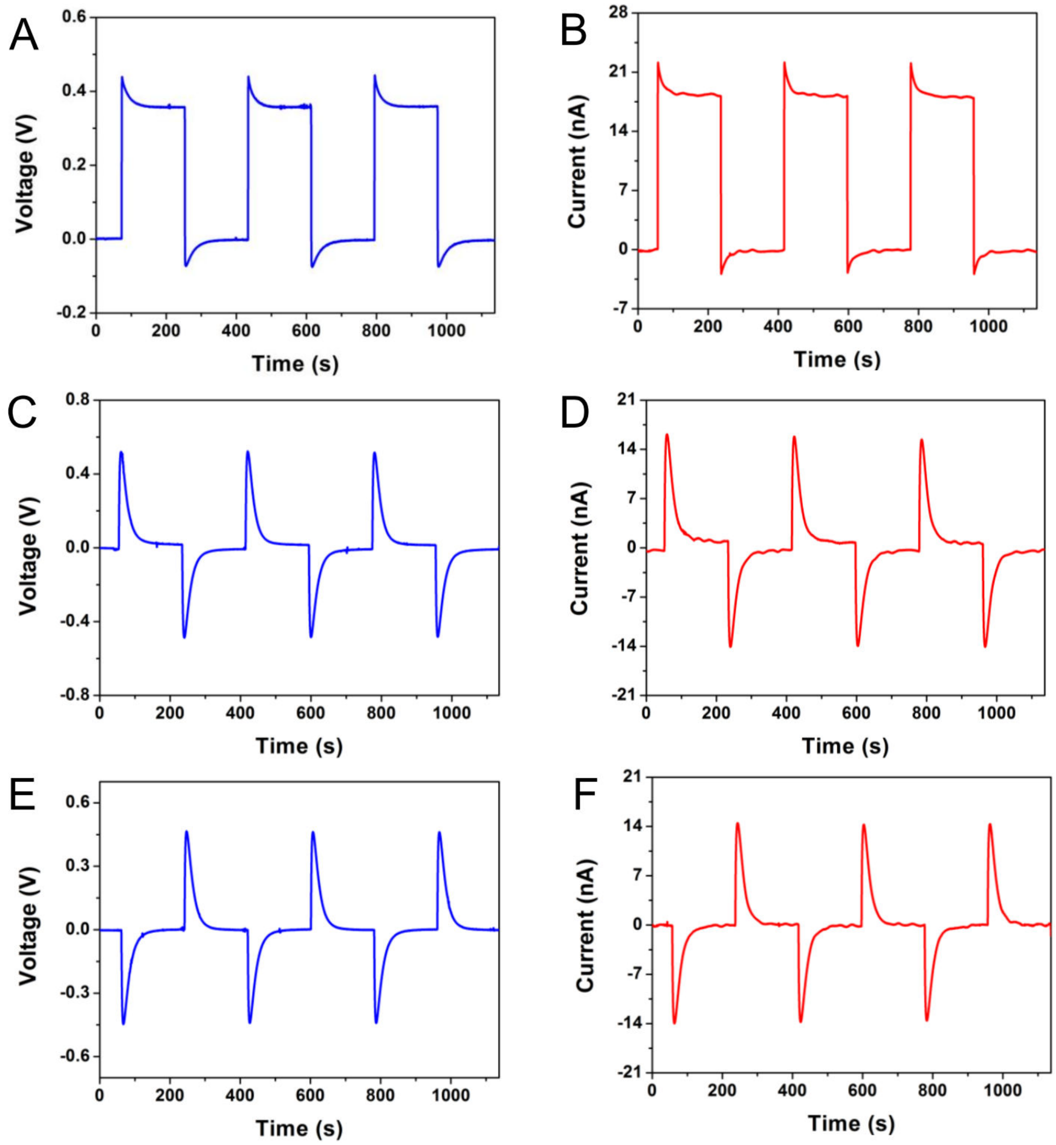

Figure S3: Measured output voltage (under a loading resistance of 50 MΩ) and output current signals of the device (ITO electrode number, N=2) at forward connection under light, heating and cooling states, related to Figure 2.

(A and B) Measured output voltage (A) and current (B) signals of the device under illumination of 365 nm light with the light intensity of 81.8 mW/cm^2.

(C and D) Measured output voltage (C) and current (D) signals of the device under a heating temperature variation with the temperature changing rate of 0.52 K/s).

(E and F) Measured output voltage (E) and current (F) signals of the device under a cooling temperature variation with the temperature changing rate of -0.44 K/s.

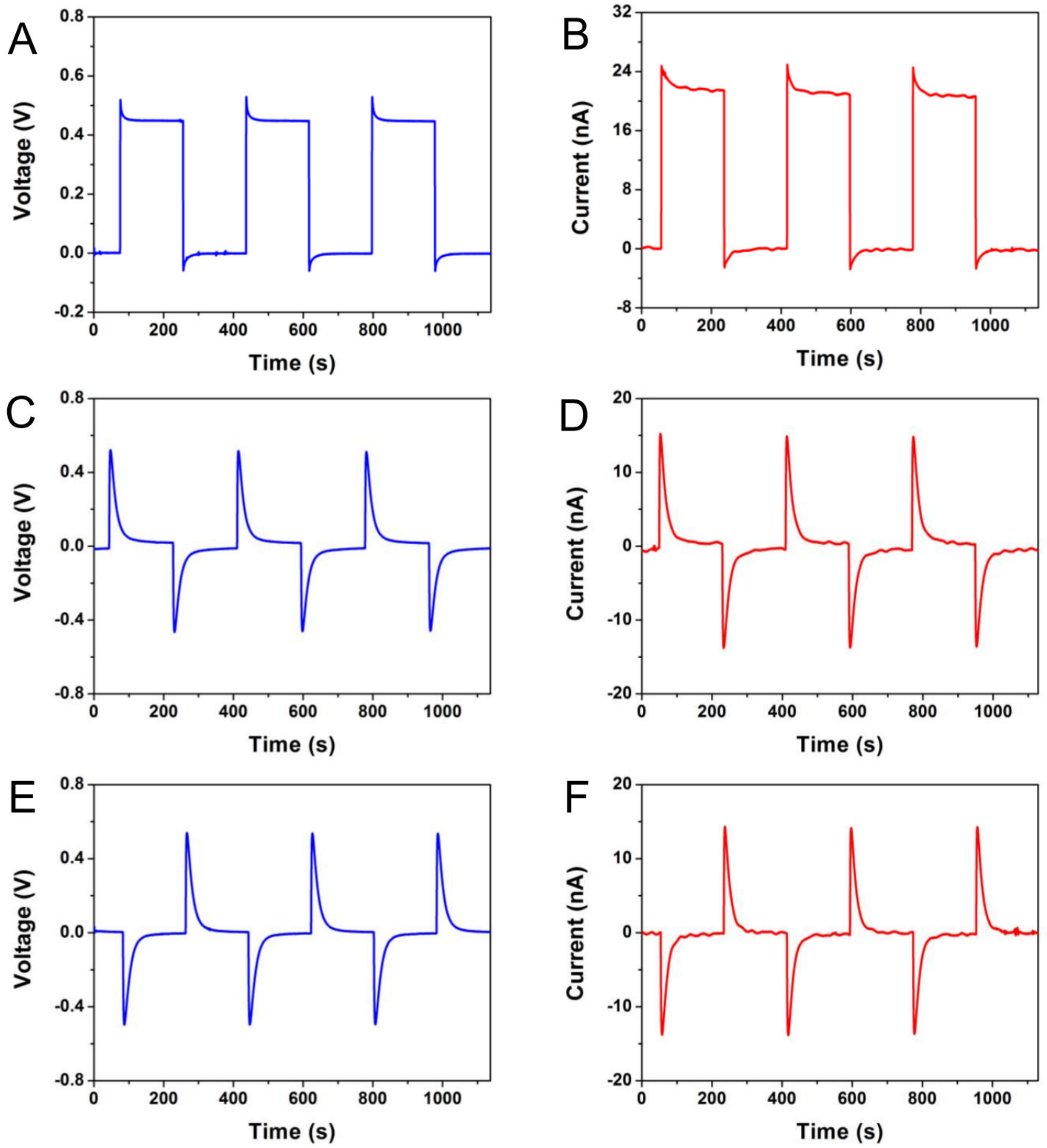

Figure S4: Measured output voltage (under a loading resistance of 50 MΩ) and output current signals of the device (ITO electrode number, N=4) at forward connection under light, heating and cooling states, related to Figure 2.

(A and B) Measured output voltage (A) and current (B) signals of the device under illumination of 365 nm light with the light intensity of 81.8 mW/cm^2.

(C and D) Measured output voltage (C) and current (D) signals of the device under a heating temperature variation with the temperature changing rate of 0.52 K/s).

(E and F) Measured output voltage (E) and current (F) signals of the device under a cooling temperature variation with the temperature changing rate of -0.44 K/s.

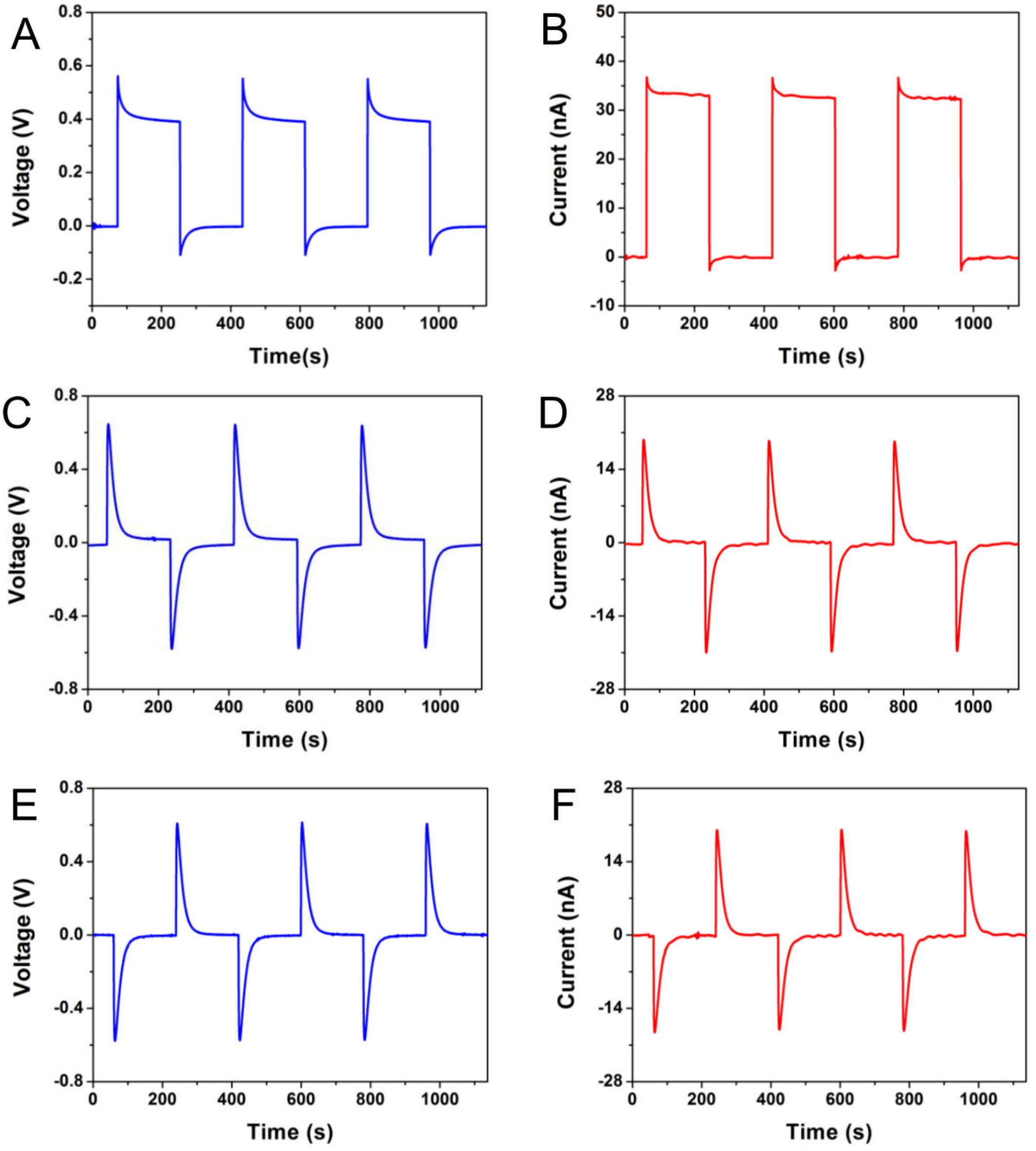

Figure S5: Measured output voltage (under a loading resistance of 50 MΩ) and output current signals of the device (ITO electrode number, N=6) at forward connection under light, heating and cooling states, related to Figure 2.

(A and B) Measured output voltage (A) and current (B) signals of the device under illumination of 365 nm light with the light intensity of 81.8 mW/cm^2.

(C and D) Measured output voltage (C) and current (D) signals of the device under a heating temperature variation with the temperature changing rate of 0.52 K/s).

(E and F) Measured output voltage (E) and current (F) signals of the device under a cooling temperature variation with the temperature changing rate of -0.44 K/s.

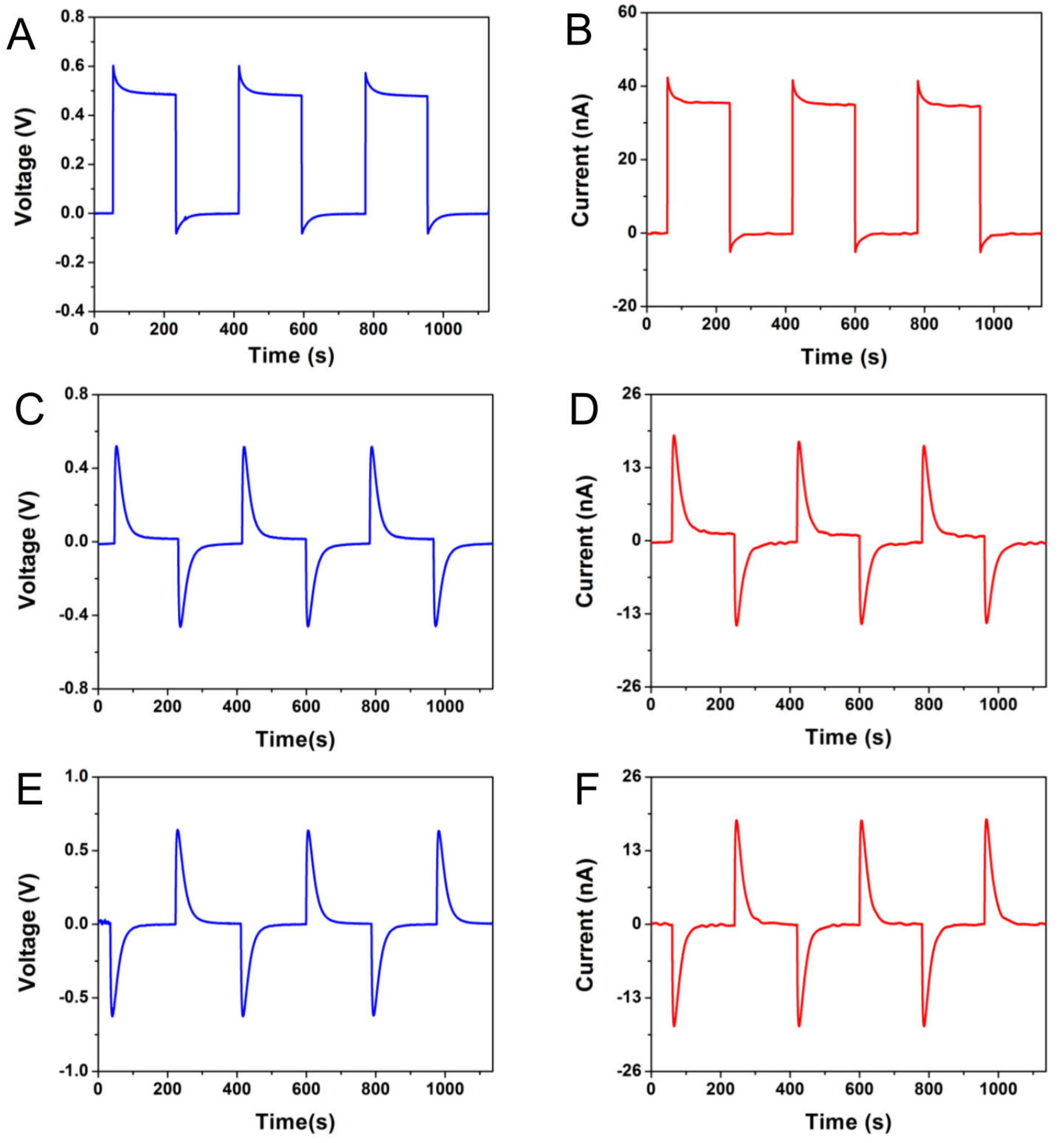

Figure S6: Measured output voltage (under a loading resistance of 50 MΩ) and output current signals of the device (ITO electrode number, N=8) at forward connection under light, heating and cooling states, related to Figure 2.

(A and B) Measured output voltage (A) and current (B) signals of the device under illumination of 365 nm light with the light intensity of 81.8 mW/cm^2.

(C and D) Measured output voltage (C) and current (D) signals of the device under a heating temperature variation with the temperature changing rate of 0.52 K/s).

(E and F) Measured output voltage (E) and current (F) signals of the device under a cooling temperature variation with the temperature changing rate of -0.44 K/s.

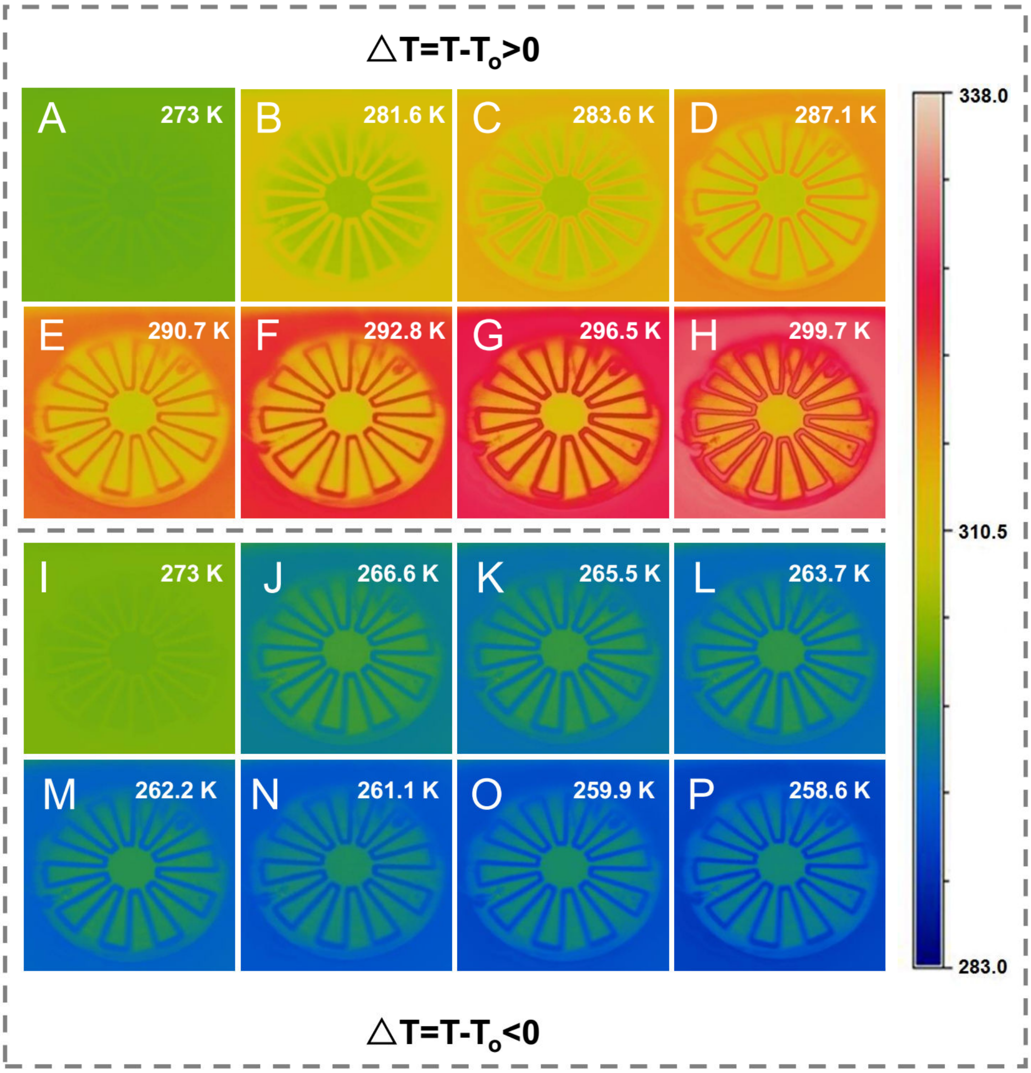

Figure S7: Infrared images of the device (ITO electrode number, N=10) at the different heating and cooling temperatures, related to Figure 2.

(A-H) Infrared images of the device under different heating states.

(I-P) Infrared images of the device under different cooling states.

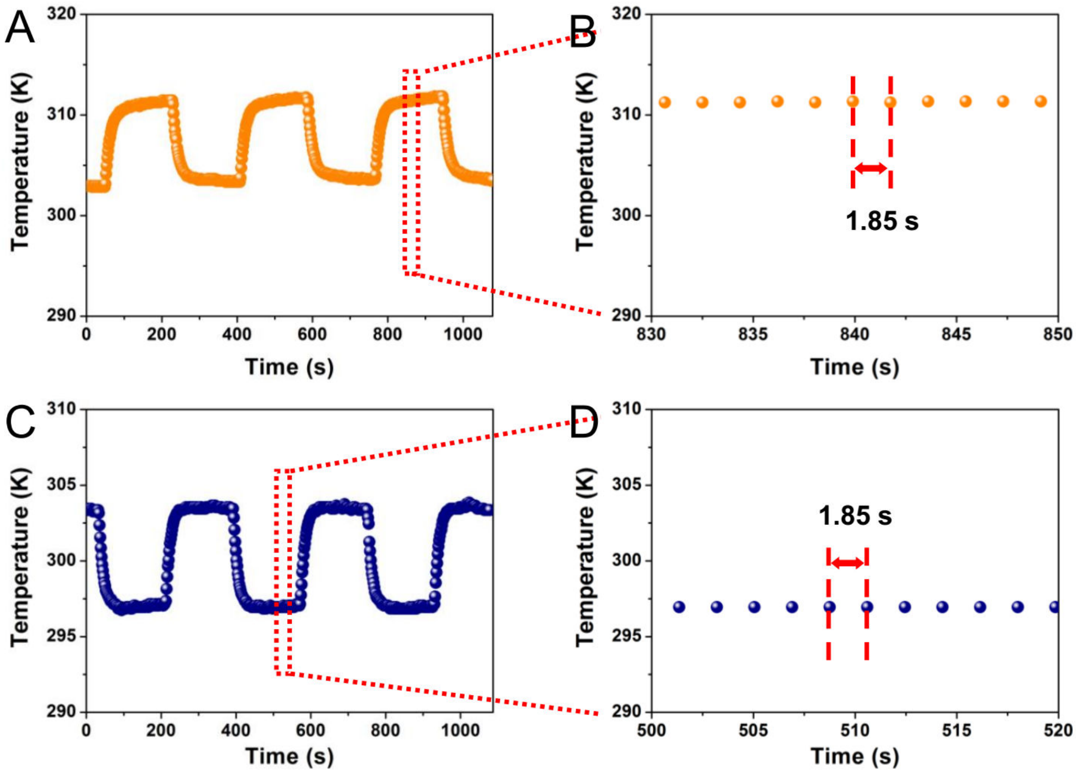

Figure S8: The cyclic temperature variations and temperature data time interval of the device (ITO electrode number, N=10), related to Figure2.

(A and B) The cyclic temperature variations (A) of the device, where the enlarged curve is displayed in (B) under a heating temperature variation condition.

(C and D) The cyclic temperature variations (C) of the device, where the enlarged curve is illustrated in (D) under cooling condition.

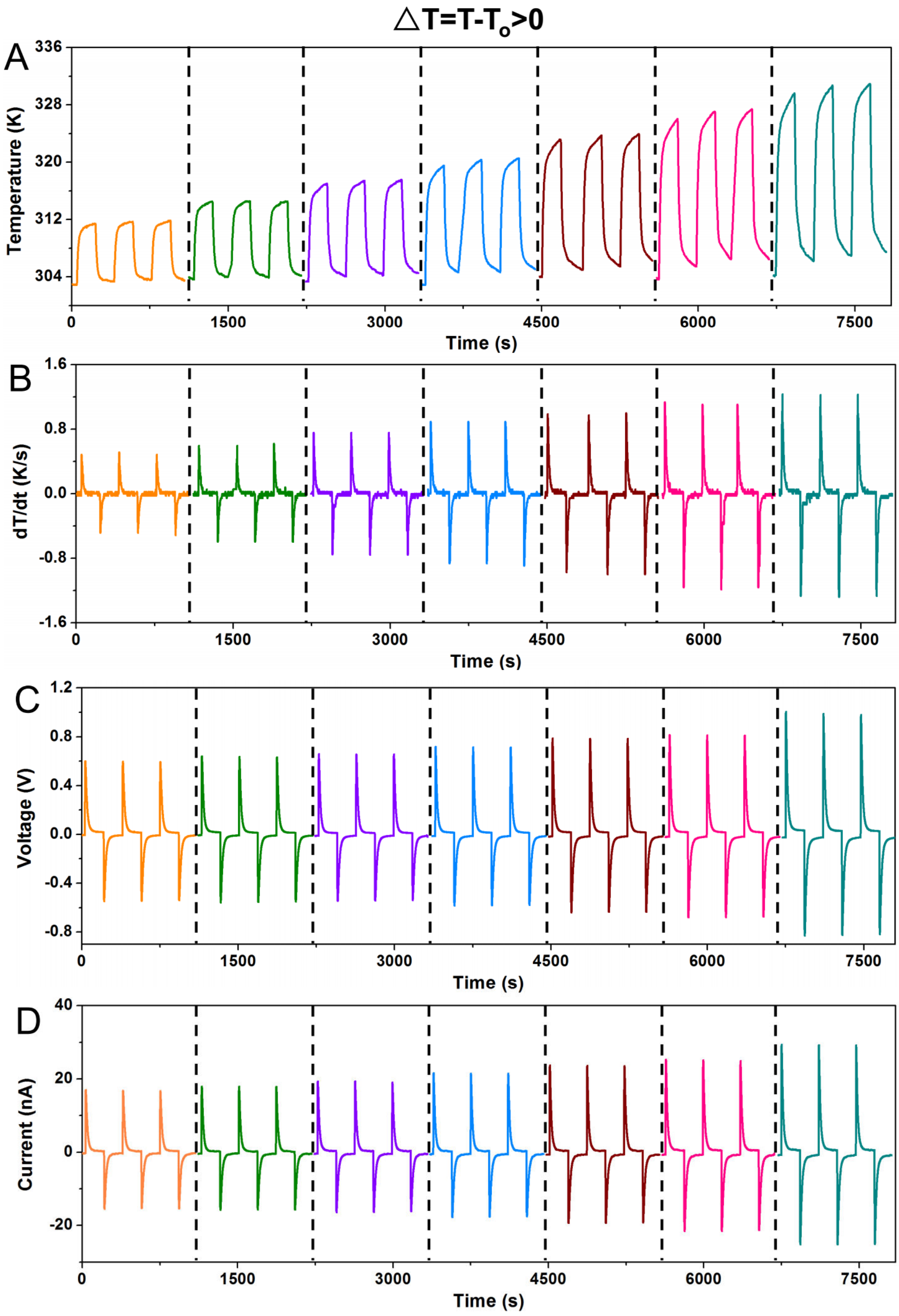

Figure S9: Heating temperature variations and pyroelectric performances of the device (ITO electrode number, N=10), related to Figure 2.

(A and B) The cyclic change in temperature (A) of the device and the corresponding differential curve (B) in the different heating conditions.

(C and D) Measured voltage (under the loading resistance of 50 MΩ) (C) and current (D) signals of the device under the corresponding temperature variations in A.

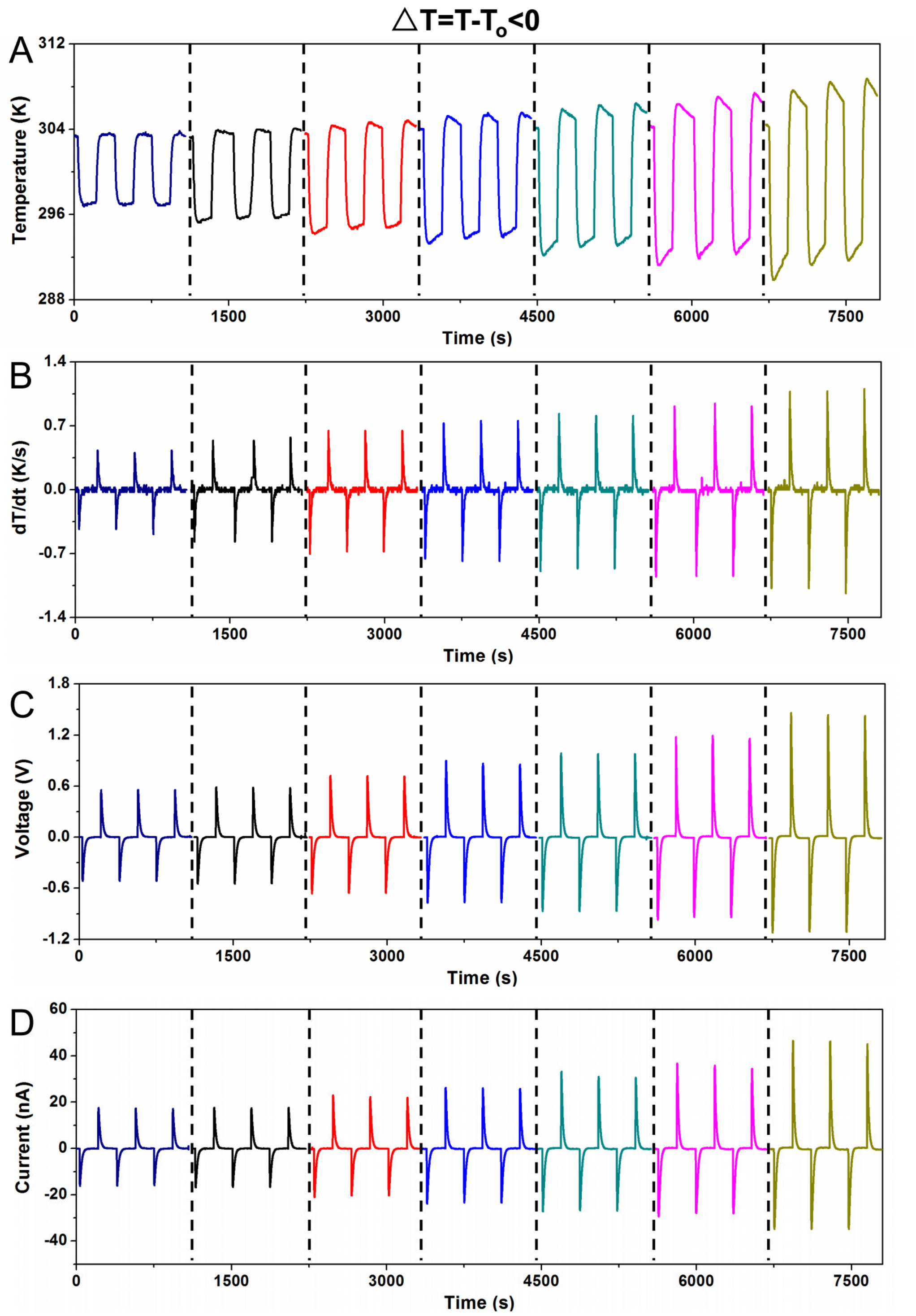

Figure S10: Cooling temperature variations and pyroelectric performances of the device (ITO electrode number, N=10), related to Figure 2.

(A and B) The cyclic temperature variations (A) of the device and the corresponding differential curve (B) in the different cooling conditions.

(C and D) Measured voltage (under the loading resistance of 50 MΩ) (C) and current (D) signals of the device under the corresponding temperature variations in A.

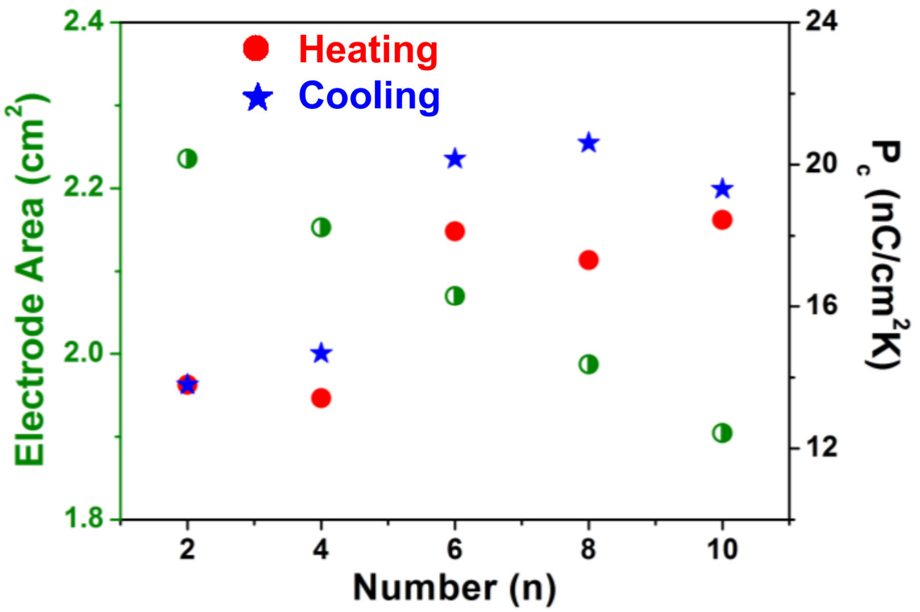

Figure S11: The effect of electrode area on the pyroelectric current coefficients of radially polarized $BaTiO_3$ device, related to Figure 2. The heating and cooling temperature changing rates of 0.52 K/s and -0.44 K/s, respectively.

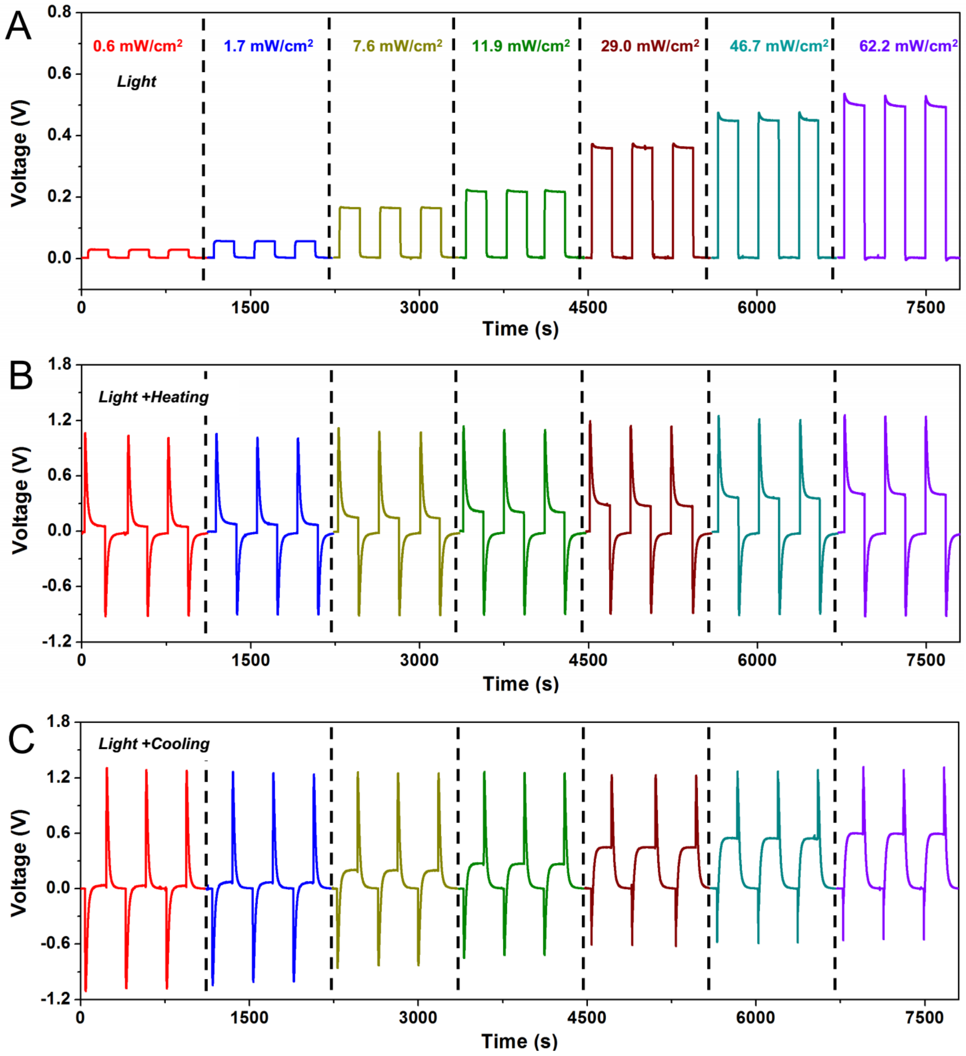

Figure S12: Measured output voltage signals of the device (ITO electrode number, N=10) under the individual light illumination, the simultaneous light illumination and heating or cooling temperature variations, related to Figure 3. (A) Measured voltage (under the loading resistance of 50 MΩ) signals of the device under 365 nm UV illuminations with different light intensities from 0.6 to 62.2 mW/cm^2.

(B) Measured voltage (under the loading resistance of 50 MΩ) signals of the device under simultaneous 365 nm UV illuminations with different light intensities from 0.6 to 62.2 mW/cm^2 and heating temperature variations with a change rate of 1.25 K/s.

(C) Measured voltage (under the loading resistance of 50 MΩ) signals of the device under simultaneous 365 nm UV LED illumination with different light intensities from 0.6 to 62.2 mW/cm^2 and cooling temperature variations with a temperature change rate of -1.08 K/s.

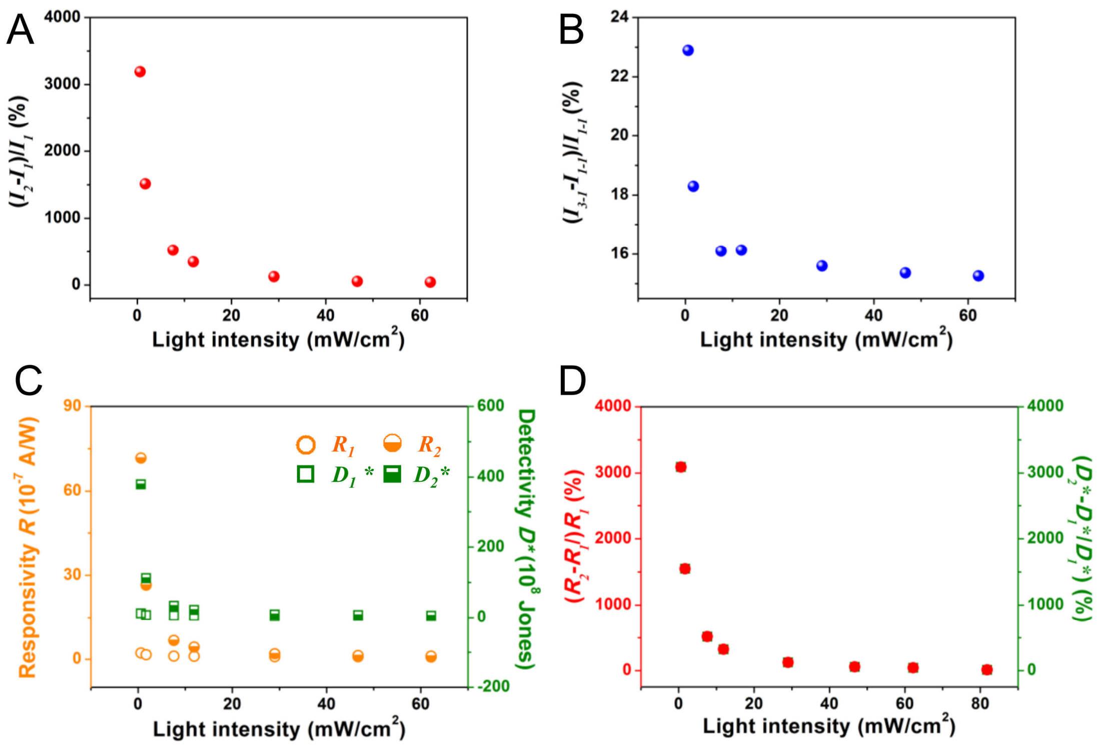

Figure S13: Photocurrent enhancement, responsivity and detectivity performances of the device (ITO electrode number, N=10), related to Figure 3. (A) Current peak enhanced ratios under the different light illumination intensities due to the ferro-pyro-phototronic effect.

(B) Stable current plateau enhanced ratios under the different light illumination intensities due to the ferro-pyro-phototronic effect.

(C and D) Responsivity and detectivity values (C) under the different light illumination intensities and the corresponding enhanced ratios (D) of responsivities and detectivities by the ferro-pyro-phototronic effect.

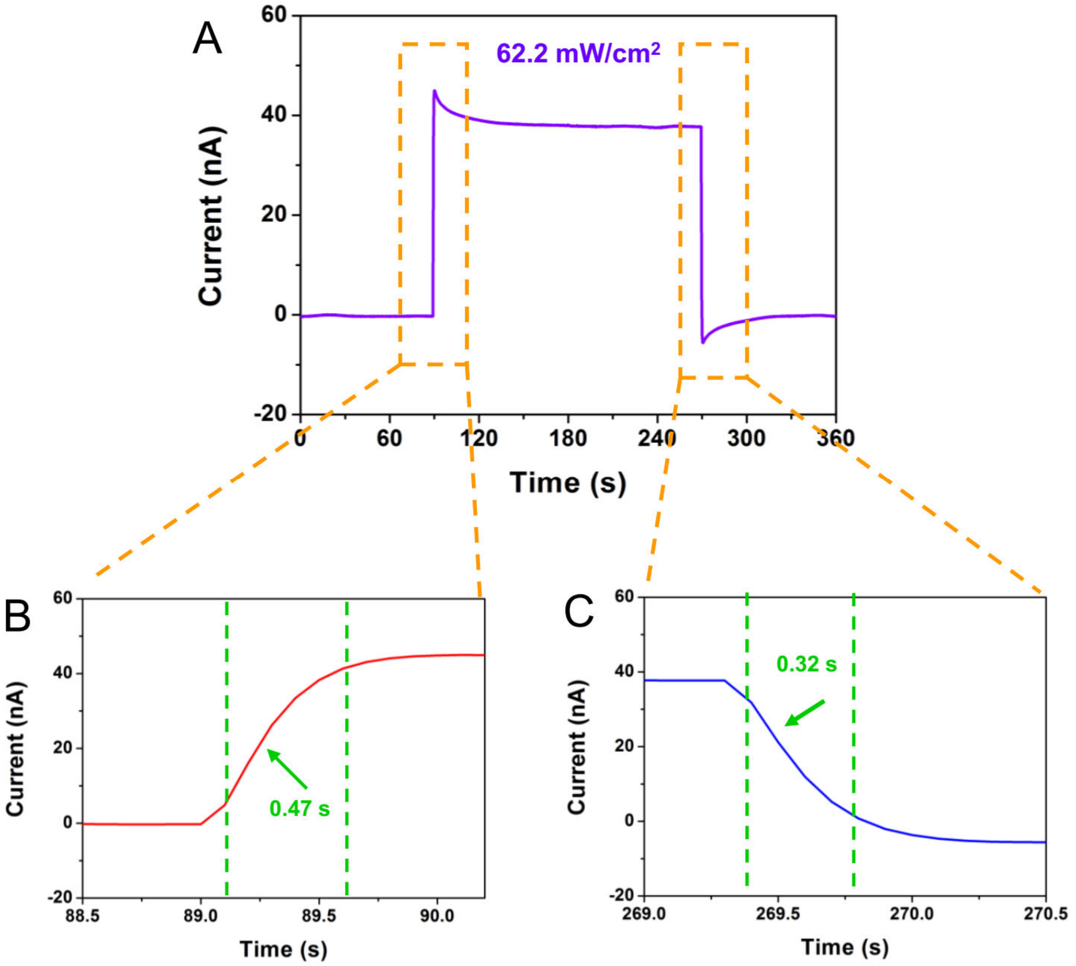

Figure S14: Response time of the device (ITO electrode number, N=10), related to Figure 3.

(A-C) The photocurrent (A) and response time including rise time (B) and fall time (C) of the device under the 365 nm illumination with light intensities of 62.2 mW/cm^2.

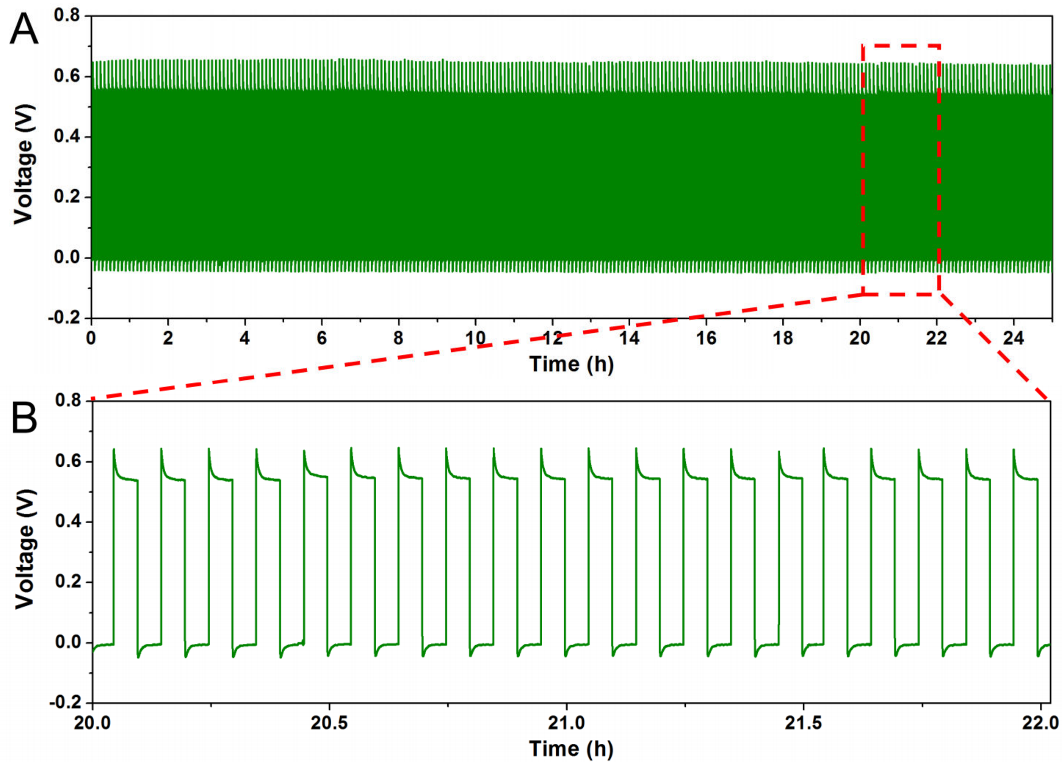

Figure S15: Stability test of the device (ITO electrode number, N=10), related to Figure 3.

(A and B) Measured output voltage signals of the device under cyclic UV light illuminations (365 nm, 81.8 mW/cm^2) for 25 hours (A), where the enlarged curve is illustrated in (B) under a loading resistance of 50 MΩ.

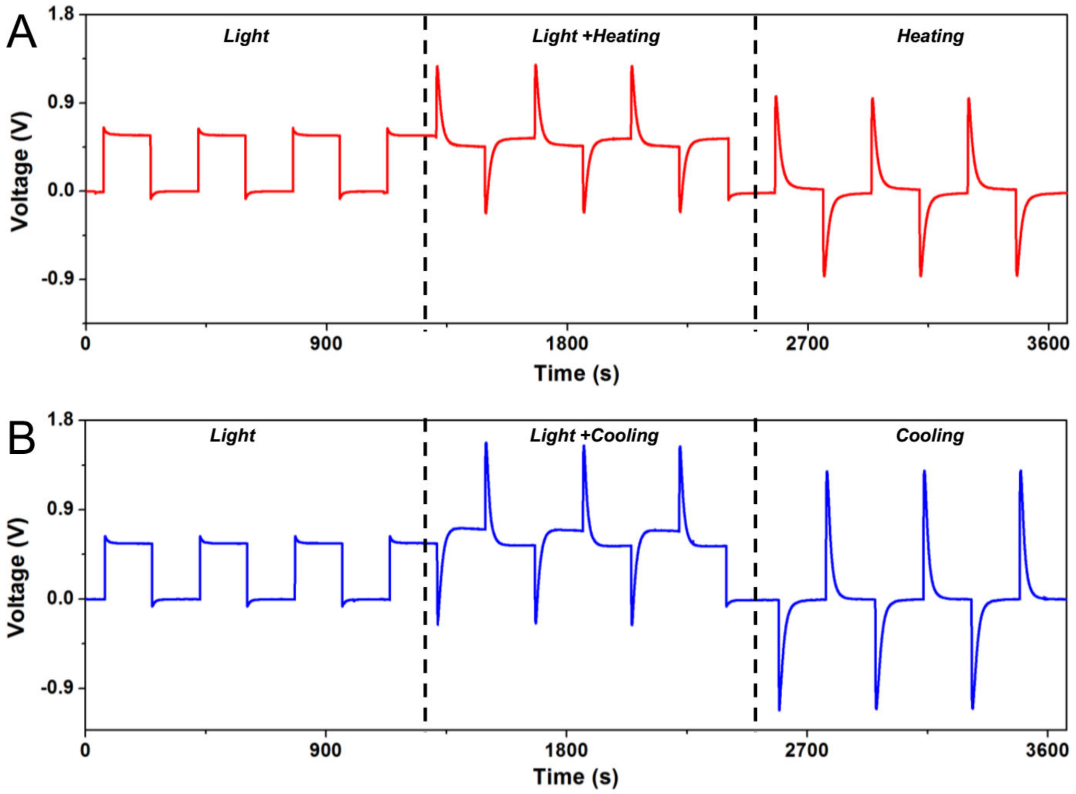

Figure S16: Measured output voltage signals of the device (ITO electrode number, N=10) under different conditions, related to Figure 4.

(A and B) Measured output voltage signals of the device under the individual light illumination, the individual heating (A) or cooling (B) temperature variations, and the simultaneous light illumination and temperature variations.

TRANSPARENT METHODS

Preparation of $BaTiO_3$ nanowires

Tetrabutyl titanate ($(C_4H_9O)_4Ti$), barium acetate ($(CH_3COO)_2Ba$) and acetyl acetone ($CH_3COCH_2COCH_3$) with a stoichiometric ratio of 1:1:2 and the total mass of 19.89 g were dissolved in 40 ml glacial acetic acid (CH_3COOH) at room temperature under a constant stirring with a magnetic stirrer for 2 h. Subsequently, polyvinylpyrrolidone (PVP, Mw ≈ 1300,000) of 1.5 g was added and stirred for 12 h. After that, the mixed solution was delivered in 20 ml plastic syringe which was connected by a stainless steel needle. A positive voltage of 12.66 kV and a negative voltage of -1.68 kV were applied between the metal needle tip and an aluminum foil collector with a distance of 10 cm. The polymer jet was ejected at a constant flow rate of 0.08 mm/min. $BaTiO_3$ nanowires were obtained by annealing the precursor nanowires at 550 ℃ for 1.5 h at the heating rate of 3 ℃/min and 1200 ℃ for 6 h in air at the heating rate of 5 ℃/min and then cooling to room temperature in the muffle furnace.

Preparation of ferroelectric $BaTiO_3$ film

The 0.3 ml PVA-water solution concentration of 2 % was added in $BaTiO_3$ nanowires of 3 g. The sample was grounded in an agate pestle and mortar to produce fine powders. And then, the obtained fine powders were transferred to a stainless steel mould that diameter of 30 mm, and it was compacted into disk-shaped sheets with approximately 30 mm diameter and 1 mm thickness at a pressure of 9 MPa with powder compression machine for 1 min. Afterwards, $BaTiO_3$ ceramic films were obtained by annealing the $BaTiO_3$ films at 650 ℃ for 1 h at the heating rate of 5 ℃/min and 1200 ℃ for 2 h in air at the heating rate of 10 ℃/min in the muffle furnace, followed by natural cooling to room temperature.

Fabrication of the devices

Firstly, a thin layer of ITO film was sputtered on the surface of the prepared $BaTiO_3$ films by a magnetron sputtering system (Beijing jinshengweina technology Co. MSP-820). Moreover, the figures were drawn using CorelDraw X4 and the engraving width is 0.6 mm, and then two radially arrayed ITO electrodes were engraved on the surface of $BaTiO_3$ film by using a laser cutting machine (Universal laser systems, PLS 4.75). Finally, the sample was poled for 30 min under an applied electric field of 2.1 kV/mm in a designed direction at room temperature.

Characterizations and Measurements

The morphology of the nanowires and nanoparticles were characterized using a field-emission scanning electron microscope (Hitachi SU8020). The crystal structure was identified by an X-ray diffractometer (Panalytical X'pert^3 powder), which using Cu Kα radiation. The temperature was measured by an infrared thermometer (Optris PI400). The output voltage and current signals of the devices were measured by a low-noise preamplifier (Stanford Research SR560) and a low-noise current preamplifier (Stanford Research SR570), respectively.
